# Utilizing Hydrothermal Processing to Align Structure and In Vitro Digestion Kinetics between Three Different Pulse Types

**DOI:** 10.3390/foods11020206

**Published:** 2022-01-12

**Authors:** Katharina Pälchen, Ben Van den Wouwer, Dorine Duijsens, Marc E. Hendrickx, Ann Van Loey, Tara Grauwet

**Affiliations:** Laboratory of Food Technology, Leuven Food Science and Nutrition Research Center, LFoRCe, Department of Microbial and Molecular Systems (M2S), KU Leuven, Kasteelpark Arenberg 22, P.O. Box 2457, 3001 Leuven, Belgium; katharina.palchen@kuleuven.be (K.P.); ben.vandenwouwer@kuleuven.be (B.V.d.W.); dorine.duijsens@kuleuven.be (D.D.); marceg.hendrickx@kuleuven.be (M.E.H.); ann.vanloey@kuleuven.be (A.V.L.)

**Keywords:** legume, hardness, structure–function, INFOGEST, starch, protein, microstructure, food structure design

## Abstract

Processing results in the transformation of pulses’ structural architecture. Consequently, digestion is anticipated to emerge from the combined effect of intrinsic (matrix-dependent) and extrinsic (processed-induced) factors. In this work, we aimed to investigate the interrelated effect of intrinsic and extrinsic factors on pulses’ structural architecture and resulting digestive consequences. Three commercially relevant pulses (chickpea, pea, black bean) were selected based on reported differences in macronutrient and cell wall composition. Starch and protein digestion kinetics of hydrothermally processed whole pulses were assessed along with microstructural and physicochemical characteristics and compared to the digestion behavior of individual cotyledon cells isolated thereof. Despite different rates of hardness decay upon hydrothermal processing, the pulses reached similar residual hardness values (40 N). Aligning the pulses at the level of this macrostructural property translated into similar microstructural characteristics after mechanical disintegration (isolated cotyledon cells) with comparable yields of cotyledon cells for all pulses (41–62%). We observed that processing to equivalent microstructural properties resulted in similar starch and protein digestion kinetics, regardless of the pulse type and (prolonged) processing times. This demonstrated the capacity of (residual) hardness as a food structuring parameter in pulses. Furthermore, we illustrated that the digestive behavior of isolated cotyledon cells was representative of the digestion behavior of corresponding whole pulses, opening up perspectives for the incorporation of complete hydrothermally processed pulses as food ingredients.

## 1. Introduction

Pulses are a sustainable and nutrient-rich food matrix being composed of different ratios of complex carbohydrates (60–65%), protein (21–25%), lipids (2–6%), and micronutrients encapsulated within (intact) cotyledon cell walls (CW) [1]. The degree to which macronutrients in pulses can be digested is highly dependent on both composition and structural features [2,3]. Various forms of processing affect the release of starch and protein from pulses and their subsequent digestion kinetics [4]. (Hydro)thermal treatment (cooking) renders pulses palatable and cotyledon cells can remain intact attenuating subsequently starch and protein digestion [5,6]. (Hydro)thermal treatment of pulses results in whole or partial starch gelatinization and progressive pectin thermosolubilization, these changes lead to the transition from cell breakage to cell separation of pulses upon the application of a force (e.g., mastication) [5,7,8,9,10]. In pre-soaked pulses, starch gelatinization occurs early in the cooking process at temperatures between 61 and 76 °C [11]. Thus, the pectin-rich middle lamellae, starch as well as the ratio between both are hypothesized to be the structure-determining components playing the most influential role in initiating the evolution from hardness to softness in pulses. 

The reductionist approach of studying digestion kinetics of the ICC fraction revealed that the preservation of cells during processing and consumption results in the CW and the starch-surrounding starch-protein matrix as physical barriers for enzymatic digestion retarding *in vitro* macronutrient digestion [3,12,13,14,15,16,17,18]. The retarded digestion kinetics are further associated with physiological *in vivo* benefits, such as low glycaemic response [19,20]. However, the digestion behavior of whole hydrothermally processed pulses, and how the characteristic microstructure influences the overall digestion kinetics have not yet been investigated. As processing is a prerequisite for the consumption of pulses and results in the (partial) transformation of the pulse architecture, the events observed during digestion will emerge from the interplay of both, intrinsic (pulse-specific) and extrinsic (process-induced) properties.

Strong evidence exists that the process-induced levels of pectin thermosolubilization determine the permeability of the CW for digestive enzymes [21,22,23], and by this modulate starch digestion (in beans) [21]. Literature suggests that CW properties are likely to govern enzymatic penetration through the CW, such as CW thickness, density, and composition, as well as the size, and the number of CW ‘pores’ [21,22,23,24,25,26,27,28]. Depending on the botanical source plant cells differ in their composition, especially their non-cellulosic polysaccharides [29], CW permeability [22] as well as starch to protein ratios [30]. As such, CW and/or starch-protein matrix might be dependent on the particular CW properties of specific botanical sources. The effect of different intrinsic boundary conditions on the CW barrier, and thus digestion kinetics of different pulse types has not been explored on equivalent levels. Besides, hydrothermal treatment has been reported to induce CW permeability, and thereby (potentially) promoting macronutrient digestion [21]. Therefore, understanding the influence of processing (conditions) on pulses’ structural properties is highly important. In this context, we hypothesize that the progressive thermosolubilization of CW pectin during thermal treatment alters the complex interaction of CW polymers and thereby increases CW permeability. Even after diffusing through the CW, the immediate enzyme-substrate encounter is hindered by an additional barrier: the co-ingested cytoplasmatic starch–protein matrix [18,24,31]. Although longer processing times were found to increase protein, and thereby starch digestion in Bambara groundnuts, the contribution of processing-induced effects on protein digestion in other pulses remains unclear [24]. Thus, there is a need for studies exploring the natural divergence between pulse types differing in these structure-determining properties on equivalent means.

Recent research in our laboratory has shown that hardness can be used as an indicator for related microstructural properties (i.e., digestibility) by monitoring the decrease of cellular adhesion of pulse cotyledon cells [24]. We hypothesize that the assessment of hardness degradation can be used as a measure for microstructural properties, more specifically the level of middle lamellae pectin solubilization, and can therefore be used for (micro)structural alignment of pulses.

The emerging interest in pulses has resulted in a major body of evidence demonstrating similarities in terms of process-induced softening (microstructure) and retarded digestion kinetics of pulses as a group [25,32,33,34] when compared to, e.g., cereals. However, most insights gained at the level of processing and digestion have been established on common beans, and ICC [5,18,28,35]. It could be questioned to what level this process-induced effect on digestive barriers can be translated to other pulse types, as one should not forget that the digestion behavior largely depends on intrinsic boundary conditions.

To understand the effects of food cellular structure on small intestinal digestion, the current study aimed to investigate the effect of hydrothermal treatment on the digestion kinetics of three different pulse types: chickpea (CP), pea (PE), and black bean (BB). The pulse types were selected based on described differences in their digestive structural barriers. The first objective was to investigate the use of hardness as a food structuring parameter to align the structural and digestion properties of the pulses. Secondly, as thermal processing times have demonstrated the capability to modulate starch digestion behavior of pulses to different extents, we aimed to investigate the effect of processing times on macronutrient digestion in ICC. So far, most insights on the engineering of pulses nutritional functionalities have been investigated on the level of ICC, being the structural characteristic with the highest level of nutrient encapsulation. Still, the majority of pulse consumption involves whole cooked pulse seed material (WSM) (i.e., cans). Therefore, the third objective of the present work focused on the evaluation of the ICC and the respective WSM of CP, PE, and BB to elucidate to what extent the digestion behavior of WSM is characterized by its characteristic microstructure.

## 2. Materials and Methods

### 2.1. Materials

Kabuli chickpeas (CP) (*Cicer arietinum*, Argentina), dry black turtle beans (BB) (*Phaseolus vulgaris*, Canada), and dry green peas (PE) (*Pisum sativum*, Europe) were harvested in 2018/2019 and obtained from Greenyard Prepared (Bree, Belgium) and Casibeans© (Melsele, Belgium) immediately after harvest. Pulse seed material was used in two ways in this work: (i) to be studied as whole seed material (WSM); and (ii) to generate individual cotyledon cells (ICC). Plant material was sorted, cleaned from defect seeds and foreign material, and kept at −40 °C until further use [31]. Storage conditions were chosen to ensure storage stability of the raw pulse seeds in their dried, low moisture state according to Kyomugasho et al. [36]. Pepsin (P7012), pancreatin (P1750), trypsin (T2011), chymotrypsin (C4129), and most other chemical reagents were purchased from Sigma Aldrich (Brussels, Belgium), except for KCl, MgCl_2_(H_2_O)_6_, NaOH, sodium potassium tartrate (Acros Organics, Geel, Belgium) NaHCO_3_, NaCl, KH_2_PO_4_ (VWR, Leuven, Belgium), and the Total Starch Kit (Megazyme, Bray, Ireland).

### 2.2. Sample Preparation

#### 2.2.1. Thermal Processing of Plant Material

**Figure 1:**
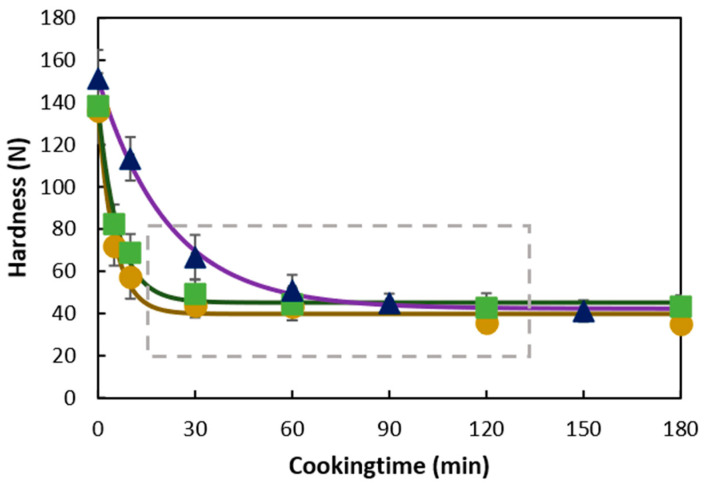
Hardness profile for chickpea (●), pea (■), or black bean (▲) whole pulse seeds cooked in demineralized water at 95 °C. Markers represent the average hardness (N) value of 25 seeds, and error bars represent the standard deviation of the measurements. Lines show fitted hardness values of the first-order fractional conversion model (Equation (4)). The grey rectangle highlights the samples selected for further *in vitro* digestion evaluations.

Dry pulse seeds were soaked overnight (16 h, 25 °C, excess of demi water) followed by hydrothermal treatment at 95 °C for 30, 60, and 90 min (CP, PE) and 60, 90, and 120 min (BB) to obtain hardness-aligned samples (Figure 1). Cooking water was discarded, and cooked pulses were mechanically disintegrated into a slurry (1 min at 8000 rpm using an IKA ultra-turrax mixer) (Janke and Kunkel, Staufen, Germany) under the addition of demineralized water (1.25:1 WSM–water *w*/*w*). Disintegrated WSM was collected, frozen, and kept at −40 °C. Seed coat%/cotyledon% ratios were determined gravimetrically as a quantitative measure for the weight distribution of the (raw) cotyledon to the whole seed material on a dry weight basis by dividing the weight of seed coat material by the weight of the cotyledon determined by weighing.

#### 2.2.2. Isolation and Characterization of ICC

To evaluate the digestion and structural properties of the ICC of CP, PE, and BB, ICC were isolated from the corresponding thermally treated WSM as described previously, with slight modifications [14,16]. In short, disintegrated, thermally treated WSM (Section 2.2.1) were wet-sieved using a Vibratory wet sieve shaker (AS200, Microtract Retsch, Haan, Germany) at an amplitude of 2.5 mm for 14 min (7 cycles of 2 min). Per pulse type, ICC were collected on an 80 μm sieve succeeded by a 125 μm sieve based on previously reported cotyledon cell sizes [31,32,33], frozen and kept at −40 °C. Homogeneity of the ICC was assessed visually (microscopy) and by laser diffraction analysis. Material captured on each sieve was evaluated to assess the yields of ICC of the three pulse types (dry basis).

### 2.3. Proximate Composition of Pulses

Dry matter content of raw and thermally treated WSM, as well as of ICC, was determined by oven drying method for 24 h at 105 °C (Thermo Fisher Scientific Inc., Waltham, MA, USA) to a constant weight in triplicate [37]. Subsequently, moisture content was calculated as percent water loss. Direct measurement of total starch content was performed in triplicate according to the AOAC Official Method 996.11 (DMSO format) using Total Starch assay Kit K-TSTA (Megazyme Inc., Bray, Ireland). Protein content was determined by automated DUMAS protein analysis system CHNS-O Elemental analyzer (CE instrument, Thermo Fischer Scientific, Waltham, MA, USA) using a conversion factor of 5.4 [38]. Total free and bound non-starch lipids were gravimetrically assessed as described previously [39]. Moreover, ash content was determined by incinerating 20 mg of dry sample for 24 h at 550 °C in a muffle furnace (Nabertherm Controller P330, Lilienthal, Germany) in triplicate. As an indirect measure for the CW content present, the fiber-rich residue (FRR) of the pulses was estimated as the percent of the remaining after all major components had been measured. The fiber-rich residue (%) was calculated by subtracting -moisture (%), protein (%), starch (%), lipid (%), and ash (%) contents of the total.

To describe the differences between the three pulses in the level of starch bioencapsulation, starch%/protein% and starch%/FRR% ratios were calculated. Starch%/protein% ratios were calculated by dividing starch by protein present as a quantitative measure for differences at the level of protein as a barrier for starch digestion. Additionally, starch%/FRR% ratios were used as a quantitative measure for the structure-determining compounds (e.g., starch, CW content).

### 2.4. Structural Characterization

#### 2.4.1. Microscopic Evaluation

Samples were suspended in demineralized water, placed on a microscope slide, and viewed with an Olympus BX-51 light microscope (Olympus, Optical Co. Ltd., Tokyo, Japan). Representative light micrographs were taken, selecting an objective of 40× magnification and cellSense Standard^®^ photo-analyzing software. Starch gelatinization was assessed using the birefringence observable in polarized light mode. To evaluate microstructural changes induced upon digestion, pellets were recovered from digests and assessed under a light microscope.

CW thickness was determined following a microscopic imaging approach on raw cotyledon cryosections. Therefore, raw, dehulled pulse cotyledons were cryosectioned (20 µm) using a cryomicrotome (Reichert Jung, Vienna, Austria). Sample sectioning was done for 30 cotyledon halves of different pulse seeds, and sections were stored in 70% ethanol (*v*/*v*) at 4 °C until analysis. Sections were washed in demineralized water to remove cytoplasmic material, stained in 0.1% calcofluor for 1 min, and subsequently washed. To examine CW thickness, stained sections were mounted onto a slide, and CW were visualized using fluorescence microscopy. The distance between 300 adjunct cells was measured at multiple spots (*n* = 1000) using cellSense^®^ software. Measured distances were halved and averaged to determine the CW thickness of one cotyledon cell. To reduce errors induced by the non-uniformity and inclination of CW, measurements were made close to the middle area of CW, and CW vertical to the cutting surface was visually chosen.

#### 2.4.2. Hardness Determination of Hydrothermally Treated Whole Pulse Seeds

To align the microstructural properties of the three different pulses, first, hardness profiles of pulses were determined using a compression test [5]. Whole, soaked pulse seeds were subjected to hydrothermal processing (Section 2.2.1) (overnight soaking; followed by 95 °C in excess of water) for varying durations (t = 0–180 min). After samples were withdrawn, processed seeds were equilibrated to room temperature for 15 min, seeds were dehulled, and a single cotyledon per seed was compressed with a cylindrical probe (25 mm) of a TA-XT2i texture analyzer loaded with a 30 kg cell (Stable Micro Systems, Godalming, UK). Hardness was measured as the maximum force (N) required to compress the cotyledon to 75% strain at a speed of 1 mm/s. Average hardness (*n* = 25) was plotted against processing time to generate a hardness evolution profile. Kinetic modeling of the hardness profile (Section 2.8) resulted in the estimated final residual hardness levels, in other words, permanent hardness (plateau). The hardness profiles of the pulses were used to obtain samples with equivalent (micro)structural properties. Hence, the three pulse types were aligned based on the processing time required to reach (residual) hardness (plateau) (grey dotted line box, Figure 1).

#### 2.4.3. Particle Size Distribution

To quantitatively determine the microstructural changes and mode of tissue failure upon mechanical disintegration of the hydrothermally treated material, volumetric particle size distributions (PSD) of raw, WSM, and ICC samples were assessed using an LS 13 320 particle size analyzer (Beckman Coulter Inc., Brea, CA, USA) equipped with a Universal Liquid Module as described elsewhere [40] in triplicate. This approach was aimed to establish a link between the macrostructural (hardness) and microstructural characteristics of the sample. Diffraction patterns of samples were converted into PSD using the Fraunhofer model. Yields of ICC were quantified by adding the volumetric fraction of particle sizes between 76 and 133 µm.

### 2.5. Thermal Properties

Thermal properties of the samples were evaluated using Differential Scanning Calorimetry (DSC) (TA Instruments, Q2000, New Castle, DE, USA) as a quantitative measurement of enthalpy and transition temperature of thermal events to estimate gelatinization parameters according to Chigwedere et al. [5] with minor modifications. Shortly, 10 ± 0.5 mg sample and 30 ± 0.5 mg water were weighed to achieve a sample to water ratio of 1:4 (*w*/*w*) in high volume DSC pans (PerkinElmer, Waltham, MA, USA), hermetically sealed and equilibrated overnight at 25 °C. Sample and reference pans (aluminum oxide) were equilibrated at 25 °C for 2 min followed by a temperature ramp (5 °C/min) from 25 to 120 °C. Data were evaluated using Version 4.5A of TA Universal Analysis 2000 Software, (TA Instruments-Waters LLC) in duplicate.

### 2.6. Static In Vitro Digestion Protocol

Hardness-aligned and mechanically disintegrated WSM and their respective ICC were digested following the standardized static *in vitro* gastrointestinal digestion protocol [41], with salivary α-amylase being substituted by demineralized water during the oral phase [31,42]. To simulate gastrointestinal digestion, all phases were performed under the addition of simulated electrolyte solutions at 37 °C in an incubator under end-over-end rotation (70 rpm). Digestion kinetics in the small intestinal phase were followed by including an individual and independent digestion tube for each pre-determined time (t = 0–180 min). At pre-determined times, digestion samples were withdrawn and enzymatic activity was immediately inactivated by heat shock (100 °C; 5 min). Digestive supernatant was separated from the pellet using centrifugation (Sigma 4–16 KS, Sigma Laborzentrifugen GmbH, Osterode am Harz, Germany) at 2000× *g* for 5 min. Both, digestive supernatant and pellet were collected, frozen, and stored at −40 °C until further analysis.

From a statistical point of view, each of the individual, single digestion reactors can be seen as a repetition [43]. Moreover, reproducibility experiments demonstrated highly reproducible results (*p* < 0.05; data not shown).

### 2.7. Quantitative Evaluation of Starch and Protein Digestion Kinetics

Starch and protein digestion kinetics of the pulse samples were studied quantitatively by assessment of the released metabolites in the digestive supernatant (Section 2.7.1 and Section 2.7.2) and qualitative observing the undigested fraction under the light microscope.

#### 2.7.1. Determination of Starch Digestion Products (%)

Digested starch (%) was determined by measuring reducing sugars present in the digestive supernatant by mixing 2 mL diluted supernatant with 1 mL dinitrosalicylic color reagent for each digestion time [44]. After incubation at 100 °C for 15 min, 9 mL of MilliQ water was added, reactants were mixed and cooled to room temperature. The absorbance of the mixture was determined (540 nm), and reducing sugars were calculated using a maltose calibration curve (0.5–2.0 mg/mL). Duplicate measurements were performed on all samples. Multiplication of the maltose concentration with 0.95 resulted in digested starch. Digested starch (%) at different digestion time points was expressed as starch equivalents of reducing sugars in the digests divided by the initial amount of starch present in the sample, expressed in percentage, see Equation (1):

(1) $$\mathrm{Digested}\;\mathrm{starch}\;\left(\%\right)=\frac{\mathrm{maltose}\;\mathrm{equivalents}\times 0.95}{\mathrm{total}\;\mathrm{starch}\;\mathrm{content}\;}\times 100$$

#### 2.7.2. Determination of Protein Digestion Products (%)

As protein digestion results in a mixture of heterogeneously sized peptides, different protein digestion evaluation approaches were used as detailed explained in our previous publication [31]. Protein digestion (%) was evaluated by measuring ‘readily bioaccessible’ and ‘digested soluble’ protein fractions [24,31] (free NH_2_ groups) using a spectrophotometric method [45,46].

To determine the ‘readily bioaccessible’ fraction, trichloroacetic acid (TCA) (3.2% final concentration) was added to the digestive supernatant precipitating larger proteins and peptides. In this way, centrifugation separates larger fractions from amino acids and small oligopeptides, which represent the potentially readily bioaccessible fraction. To describe the digested protein by its monomeric constituents (amino acids), the digestive supernatant was hydrolyzed by HCl (6 N) at 110 °C for 16 h followed by rotary evaporation of the acid as explained in detail elsewhere (‘digested soluble protein’) [24]. This allows quantifying the degree to which protein has been solubilized and could leave the cell due to enzymatic degradation.

Free NH_2_ groups in the protein fractions were determined by OPA spectrophotometric assay. To determine the total α-amino groups in the undigested sample ($NH_{2\;\left(total\right)}$), the undigested sample ($NH_{2\;\left(initial\right)})$ corresponding to the oral phase (2 min) or the digested soluble protein fraction ($NH_{2\;\left(hydrolyzed\right)})$, respective amounts of the sample or digestive supernatant (5 mg, 0.5 mL, 0.5 mL) were hydrolyzed with 1 mL HCl (6 N) at 110 °C for 16 h, in duplicate. After acid removal, samples were diluted in Milli-Q water and filtered (0.25 µm pore size). OPA reagent was freshly prepared for each experiment, as described by Zahir et al. [46]. 3 mL OPA reagent were mixed with 400 µL sample (Milli-Q water (blank), L-serine (standards), or digestive supernatant). The mixture was incubated in the dark for 2 min at room temperature, and absorbance at 340 nm was evaluated. The concentration of free α-amino groups was calculated as L-serine equivalents (12.5–100 mg/L). Digested protein (%) was calculated and expressed as ‘digested soluble protein’ as shown in Equation (2) and ‘readily bioaccessible protein’ (Equation (3)). Measurements were performed in duplicates.

(2) $$\mathrm{Digested}\;\mathrm{soluble}\;\mathrm{protein}\;\left(\%\right)=\frac{NH_{2\;\left(hydrolyzed\right)}-NH_{2\;\left(initial\right)}}{NH_{2\;\left(total\right)}}\times 100$$

(3) $$\mathrm{Readily}\;\mathrm{bioaccessible}\;\mathrm{protein}\;\left(\%\right)=\frac{NH_{2\;\left(TCA\right)}-NH_{2\;\left(initial\right)}}{NH_{2\;\left(total\right)}}\times 100$$

### 2.8. Data Analysis: Kinetic Modeling and Statistical Analysis

Kinetic modeling of experimental data was carried out using SAS version 9.4 (SAS Institute, Inc., Cary, NC, USA). Hardness evaluation, starch, and protein digestion kinetics in the small intestinal phase were modeled using a fractional conversion model (Equation (4)), previously used to describe hardness degradation [5,21,25,42] and *in vitro* starch and protein digestion in pulses [24,31]. Using kinetic modeling, the reaction rate constant of digestion or hardness decay (k (min^−1^)) and the final digested starch or protein concentration or final hardness (*C_f_*) were estimated:

(4) $$C_{t}=C_{f}+\left(C_{i}-C_{f}\right)\times e^{-kt}$$

where$\;C_{i}$ and $C_{t}$ are hardness value or digested starch or protein (%) at 0 min and t min cooking or digestion, respectively; $C_{f}\;$is the residual hardness or final concentration of digested starch or protein at extended cooking or digestion times; and k denotes the estimated reaction rate constant (min^−1^). The fit of the model was evaluated by calculating R^2^_adjusted_ and visual analysis of the residual and parity plots. Initial reaction rates were calculated by determining the slope of the tangent line to the fractional conversion model at the start of the reaction (t = 0 min) as earlier described by Pallares Pallares et al. [17]. Significant differences were assessed by the use of their 95% confidence intervals. To verify significant differences or similarities among the estimated kinetic parameter (k and $C_{f}$), joint confidence region analysis (JCR) (α = 0.05) was performed using Equation (5). By constructing JCR, the correlation between the simultaneously estimated parameters was considered.

(5) $$SSE=SSE\;\left(\theta \right)\left(1+\frac{p}{n-p}\;F\left(p,n-1,1-\alpha \right)\right)$$

Additionally, after a model discrimination procedure, starch digestion of black beans during the small intestinal phase was modeled using a logistic model (Equation (6)) suitable to describe the characteristic initial lag phase, recently used to describe starch digestion kinetics in common bean and Bambara groundnut ICC [21,24,47].

(6) $$Starch_{t}=\frac{Starch_{f}}{1+e^{\left[\frac{4\times k_{max}}{Starch_{f}}\left(\lambda -t\right)+2\right]}}$$

where $Starch_{t}\;$is digested starch (%) at time *t*, $Starch_{f}$ is the plateau digested starch (%), $k_{max}$(%starch/min) is the reaction rate constant, and λ (min) is the duration of the lag phase.

Data from proximate analysis, thermographs, PSD, and DSC were statistically analyzed byTukey HSD post hoc test (*p* ≤ 0.05) using JMP Pro 15 (SAS Institute, Cary, NC, USA).

## 3. Results

### 3.1. Hardness Evolution of Chickpea, Pea, or Black Bean during Hydrothermal Treatment

Hardness profiles showing the characteristic tissue softening of CP, PE, and BB as a function of hydrothermal processing at 95 °C are illustrated in Figure 1. The hardness of all pulses decreased with increased processing times, and the profile was characterized by two distinct domains: (i) a period in which hardness decreased; followed by (ii) a period in which hardness reached a plateau (residual hardness = 42 ± 3 N), which was characterized by the absence of a process-induced effect on hardness (CP/PE ≥ 30 min; BB ≥ 60 min).

Kinetic modeling using a fractional conversion model was employed (Supplementary Materials Table S1) to quantitatively describe the hardness profile, residual hardness (*H_f_*), and the reaction rate constant (*k*) for softening. Overall, significant differences in the reaction rate of softening, *k,* for all three pulse types were observed. *k* was significantly lower for BB (0.046 ± 0.002 min^−1^) as compared to CP and PE implying longer processing times required to reach softening. Whereas CP and PE required 30 min to soften completely, BB softening required twice as long. Irrespective of the different softening rates, the three pulses reached similar final hardness levels, which corresponded to 70 ± 2% hardness reduction upon hydrothermal treatment in all pulses.

### 3.2. Microstructural Changes as a Result of Hydrothermal Treatment

**Figure 2:**
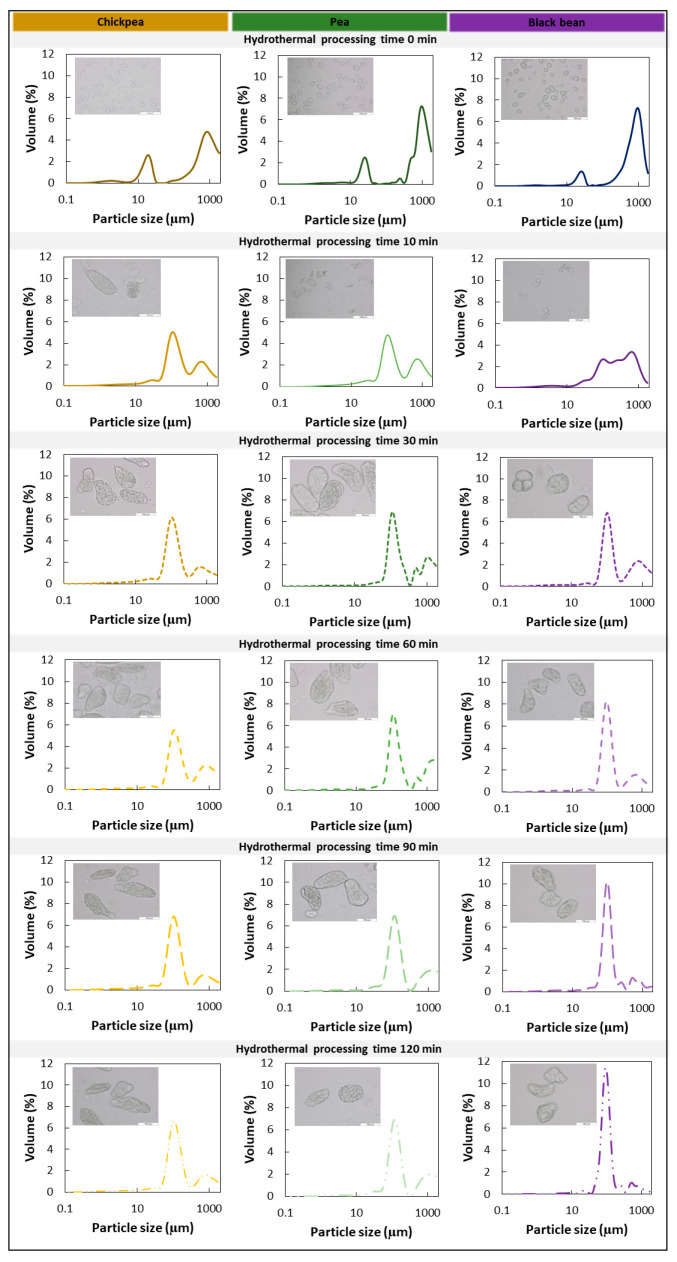
Volumetric particle size distributions and representative microscopic images (scale bar: 100 µm) of whole seed material from hydrothermally treated and mechanical disintegrated chickpea (yellow), pea (green), or black bean (purple) using different processing times (95 °C, t: 0–120 min). Means of triplicate evaluations are presented.

To explore the process-induced microstructural properties of cooked seeds, the microstructure of mechanically disintegrated pulses (WSM) was assessed qualitatively (microscope) and quantitatively (PSD) as a function of processing time (Figure 2) to establish their tissue failure mode(s). Figure 2 shows micrographs and PSD of disintegrated WSM. For all three pulses, the visual and quantitative evaluation revealed similar microstructural properties as a function of processing time as described previously for common beans [5]. Microstructural properties for all pulses were characterized by the evolution from free intracellular material (starch granules, CW material) in soaked pulses to a co-existence of free starch granules along with ICC with increasing processing times (CP = 10 min; PE = 15 min, BB = 20 min). Further cooking for 30 or 60 min and beyond yielded predominantly ICC (CP, PE = 30 min; BB = 60 min).

**Figure 3:**
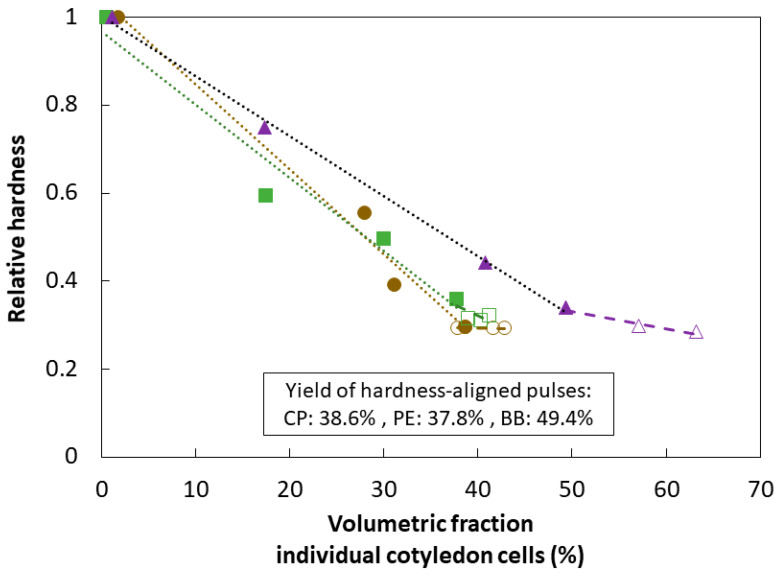
Quantitative evaluation of the individual cotyledon cell fraction determined by particle size distribution as a function of the relative residual hardness after processing for chickpea (CP) (●), pea (PE) (■), or black bean (BB) (▲) material. Means of triplicate evaluations are presented. Filled symbols (●) are linked to the time domain for which hydrothermal processing resulted in hardness decay. Open symbols (○) are linked to the time domain for which extended hydrothermal processing did not further affect the hardness level (e.g., plateau region). Dotted lines show fitted linear trendlines for each of the two domains (i.e., hardness decay versus hardness plateau).

Figure 3 depicts this increased yield of ICC quantitatively as a function of hardness decrease as well as for the hardness plateau induced by hydrothermal processing. For CP and PE, the ICC fraction did not increase further after reaching residual hardness values. Contrary, for BB, the volumetric fraction of ICC (%) increased despite having reached the hardness plateau (BB60 = 49%, BB90 = 57%, and BB120 = 63%).

### 3.3. Macronutrient In Vitro Digestion Patterns of Hardness-Aligned Pulses

By controlling the passage of digestive enzymes and hydrolysis products, pulses’ structural features (i.e., tissue failure mode) determine the digestive responses [2]. To be able to compare digestive properties of different pulse types on (more) equivalent structural levels, we employed (residual) hardness as a food structuring parameter to anticipate characteristic tissue failure modes and align (micro)structural properties. Therefore, processing times were chosen to represent the earliest cooking times resulting in residual hardness levels (42 ± 3 N) (Figure 1), at which ICC form the characteristic microstructure (Figure 2) (see Section 3.1 and Section 3.2). First, starch and protein digestion kinetics of ICC isolated from hardness-aligned CP, PE, and BB, after cooking for 30, 30, and 60 min, respectively, were evaluated (Section 3.3.2 and Section 3.3.3). In a second step, the digestive behavior of the respective WSM was assessed and compared to the corresponding ICC (Section 3.3.4).

#### 3.3.1. Proximate and Structural Characterization of Hardness-Aligned Pulses (ICC and WSM)

**Figure 4:**
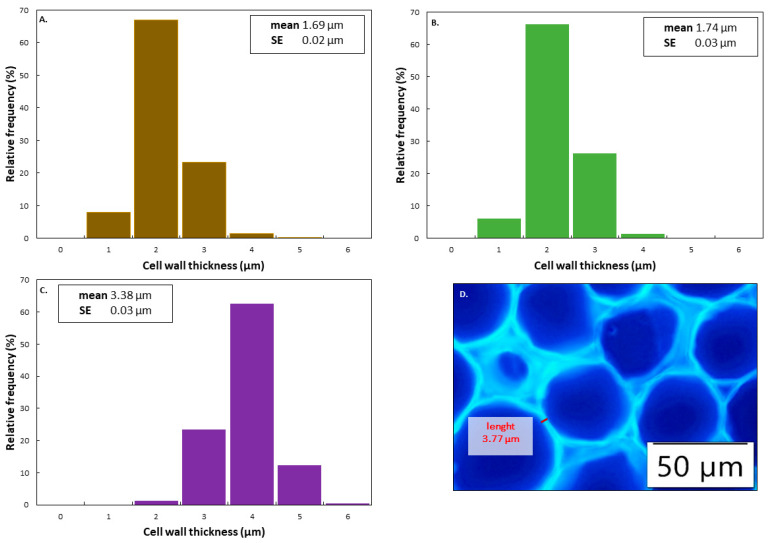
Histograms of measured cell wall thickness distribution from cryosections of raw chickpea (yellow) (**A**), pea (green) (**B**), or black bean (purple) (**C**) seeds. Exemplary cryosection of chickpea cotyledon stained with 0.1% calcofluor indicating the region (in red) that was considered for the thickness measurement (scale bar: 50 µm) (**D**).

**Proximate characterization:** Analysis of starch (33–61%), protein (17–21%), lipid (2–7%), and ash (2–4%) confirmed previously reported differences in chemical composition between pulses for both sample types (WSM; ICC) (Table 1) [15,32,48]. The calculated FRR content in the raw pulses ranged from 27 to 33%. Whilst all three pulse types showed comparable moisture and protein contents, BB had a significantly lower starch content. These differences resulted in different starch%/protein% ratios (BB < CP, PE). Moreover, BB was characterized by the lowest starch%/FRR% ratio. Along with the highest FRR, BB exhibited the highest CW thickness of 3.38 µm, being twice as thick as for CP and PE (Figure 4). Figure 4D shows a stained cryosection and includes an indication of the region of measurement. Additionally, while for CP and PE, the seed coat fractions accounted for 12.3% and 13.1%, respectively, the seed coat of BB made up 10.3% of the whole raw seed. Overall, comparing the intrinsic characteristics between the three pulses, BB demonstrated more distinct characteristics in terms of structure-governing components (CW and starch content) as compared to CP and PE.

**Figure 5:**
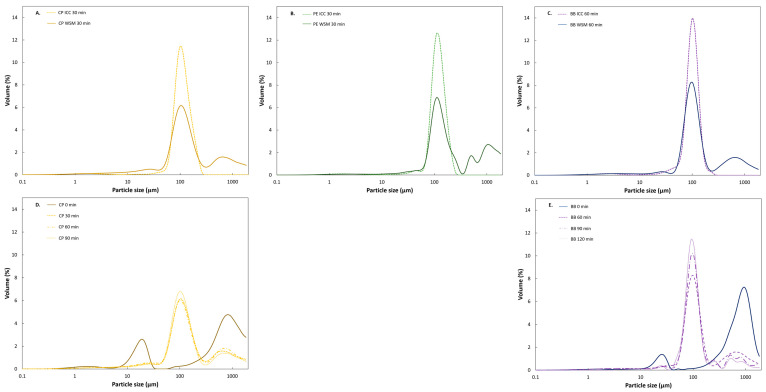
Particle size distribution of hardness-aligned pulses (**A**–**C**) and pulses subjected to varying hydrothermal processing times (**D**,**E**). Means of triplicate evaluations are presented. Pulses were hydrothermally processed, followed by mechanical disintegration. For hardness-aligned chickpea (CP) (●) (**A**), pea (PE) (■) (**B**), and black bean (BB) (▲) (**C**) particle size distributions of whole seed material (WSM) and thereof wet-sieved individual cotyledon cell (ICC) fraction are displayed. Particle size distribution of the raw and hydrothermally treated whole seed material for chickpea (**D**) and black bean (**E**) are displayed to study the effect of hydrothermal processing (95 °C for 0, 30, 60, 90, or 120 min) on microstructural and digestive properties.

**Structural characterization***:* We employed hydrothermal processing to residual hardness to obtain pulses with similar microstructural characteristics. The microstructural properties of processed pulse samples were similar, which became apparent when looking at the PSD (Figure 5) and the micrographs. PSD and microscopic observations of ICC fractions of hardness-aligned samples were characterized by unimodal distributions and the abundance of majorly ICC. On average, ICC isolated from BB exhibited the smallest (104.66 ± 0.21 µm) and PE the largest (124.84 ± 0.08 µm) particle sizes. CP and PE cells were elliptically shaped, which translated into a wider PSD and average mean sizes for both. BB cells typically showed spherical shapes resulting in a more homogeneous and narrower PSD. The PSD of the different sample types (WSM versus ICC) revealed structural differences no matter the pulse type (Figure 5). For all pulses, higher heterogeneity in terms of particle size was reflected in the multimodal PSD of WSM, ICC were identified as the characteristic fraction next to free material (starch granules, protein bodies) and bigger cells and clusters, mainly originating from the seed coat. Hence, hydrothermal processing to the residual hardness levels yielded ICC for all three pulses (WSM and ICC).

As a result of the preceding hydrothermal treatment, starch gelatinization, despite encapsulation, was visually confirmed by the absence of birefringence using polarized light microscopy. Additional insights on the gelatinization status of samples were obtained by DSC thermographs confirming complete starch gelatinization with residual enthalpies of below 0.2 J/g in all cases (Supplementary Materials Table S2).

#### 3.3.2. Starch Digestion of ICC Fraction of Hardness-Aligned Pulses

**Figure 6:**
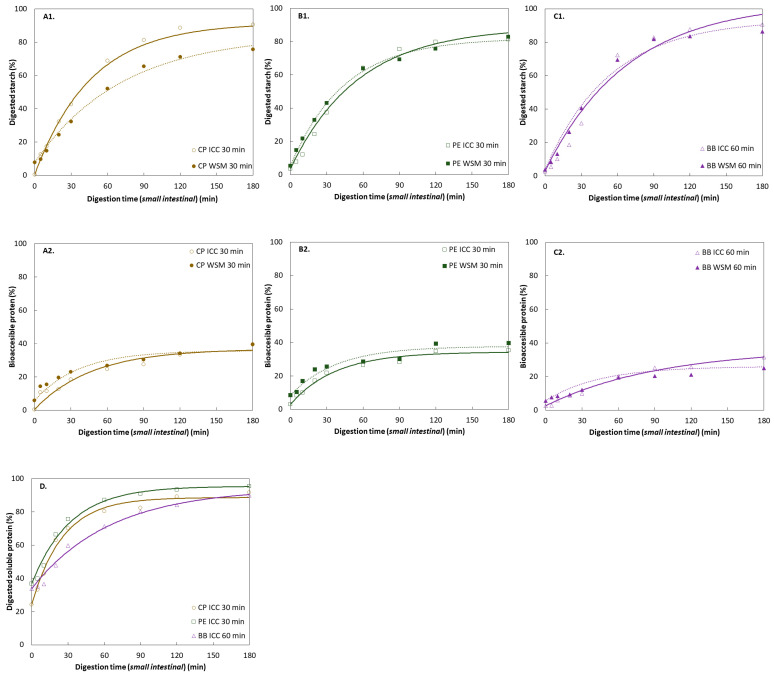
*In vitro* starch (**A**–**C1**) and protein (**A**–**C2**,**D**) small intestinal digestion kinetics of individual cotyledon cells (ICC) (○ open symbols) and whole seed material (WSM) (● closed symbols) from hardness-aligned chickpea (CP) (●) (**A**), pea (PE) (■) (**B**), or black beans (BB) (▲) (**C**) hydrothermally treated for 30, 30, or 60 min, respectively. Symbols represent experimental values, while lines represent values predicted by a fractional conversion model (Equation (4)).

**Figure 7:**
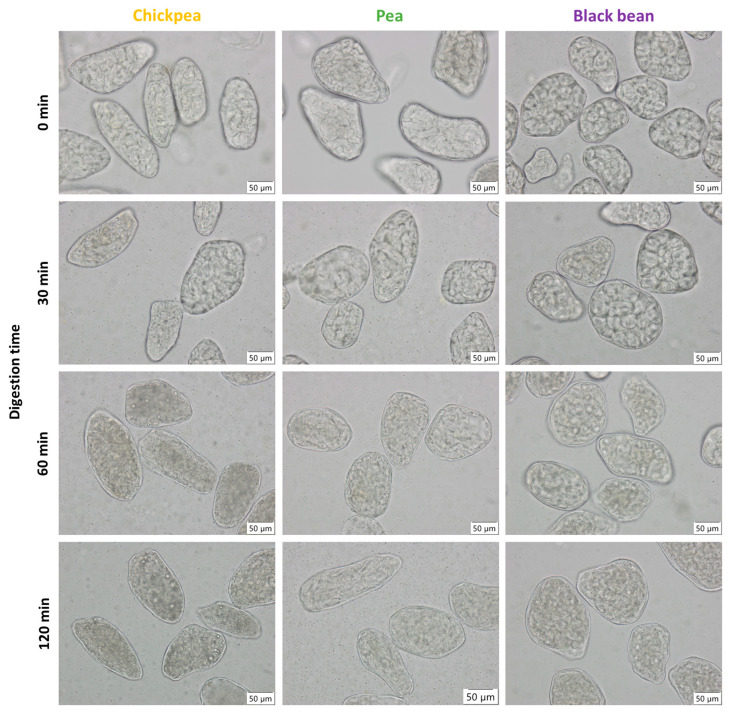
Representative microscopic images (scale bar: 50 µm) of individual cotyledon cells (ICC) recovered after digestion (t = 0–120 min). Images were taken as a function of the *in vitro* simulated small intestinal digestion phase. Samples are cotyledon cells isolated from chickpea (CP), pea (PE), or black bean (BB) hydrothermally treated for 30, 30, or 60 min, respectively.

In pulses, carbohydrates are the major macronutrient fraction, mainly consisting of starch (35–60%), non-starch polysaccharides, and to a lesser extent, oligosaccharides [49]. ICC samples of hydrothermally treated CP, PE, and BB WSM were generated and subsequently subjected to *in vitro* digestion simulations. (Figure 6). The changes during digestion were qualitatively assessed by microscopic visualization of the pellets under a light microscope (Figure 7).

Experimental data showed for the hardness-aligned pulses an initial period where starch digestion increased as a function of time, which was followed by the attainment of almost stable digestion levels (t = 90–180 min). All three ICC fractions reached similar extents of starch digestion (81–91%). Progression of starch digestion was quantitatively evaluated by kinetic modeling using a fractional conversion model (Equation (4)) to estimate final levels of starch digestion (*starch_f_*, %) and reaction rate constant (*k*, min^−1^) as previously described for other pulses (Table 2) [17,25,31].

Supplementary Materials Figure S1A shows the 95% JCR for the jointly estimated parameters. The overlapping joint confidence regions for all samples illustrate the overall similar digestion behavior of the three hardness-aligned pulses. Notwithstanding, zooming in into the digestion evolution within the first 30 min, experimental data of BB showed a slower progression of starch digestion, indicating the presence of a lag phase. In previous work on (common) beans, this lag phase was linked to the CW barrier effect for starch digestion [21,24]. Contrarily, a lag phase was not observed for CP and PE. Due to the presence of the lag phase for BB, a logistic model, as previously employed for other pulse types [21], was used to model the data resulting in an estimate of the lag phase of λ = 11 min (Supplementary Materials Table S3). When the logistic model was applied to the CP and PE data, no meaningful estimates for the lag phase were obtained (data not shown). Consequently, to allow comparison a common empirical model equation (fractional conversion model) was used to model data of all pulses. However, through the calculation of the initial reaction rate constant using the fractional conversion model, differences in the initial digestion kinetics (presences versus absence of a lag phase) could be identified between BB versus CP and PE. 

#### 3.3.3. Protein Digestion of ICC Fraction of Hardness-Aligned Pulses

In addition to the CW barrier, the co-encapsulated intracellular protein matrix has been described to form a barrier for starch digestion, thereby attenuating starch digestion [23,24,31]. Moreover, the protein fraction forms the second-largest macronutrient fraction in pulses (~20%). To explore this barrier effect, starch to protein ratios (starch%/protein%) were assessed for all samples (Table 1). A lower starch to protein ratio indicates more protein surrounding starch. Consequently, a more densely protein-packed intracellular matrix might exert an increased barrier effect for starch, retarding enzyme-substrate interaction more pronounced. For all sample types (raw, WSM, and ICC), BB showed the lowest starch to protein ratio, followed by CP and PE.

Protein digestion products were evaluated as a function of small intestinal digestion time, both at the level of ‘readily bioaccessible protein’ (TCA) (Figure 6A2,B2,C2) and ‘digested total soluble protein’ (full hydrolysis) (Figure 6D). Common for both evaluation approaches, upon entering the small intestinal phase, the formation of free α-amino groups increased due to the proteolytic activity of small intestinal proteases. The data were modeled using a fractional conversion model and estimated kinetic parameters are displayed in Table 3. Both evaluation approaches were characterized by periods in which protein digestion increased as a function of time (~t = 60 min), followed by periods where protein digestion not further increased.

**Digested soluble protein:** At the beginning of the small intestinal digestion, about 25–37% of protein was already solubilized (Figure 6D). Experimental data showed that gastric protein digestion of PE resulted in the highest amount of protein solubilized, followed by BB and CP. Differences in the initial solubilized protein (t = 0 min) between the pulses suggest that different amounts of protein had been solubilized upon gastric proteolysis. As small intestinal digestion progressed, the solubilization of protein continued until leveling off after 60 min (CP and PE) or 90–120 min (BB). The differences in estimated kinetic parameters and initial reaction rate, being the highest for CP, suggest faster protein hydrolysis in CP. Interestingly, CP initially showed the lowest amount of soluble digested protein (t = 0 min). Similar to BB starch digestion, a lag phase of ~10 min was observed for BB protein digestion. Nonetheless, no significant differences in overall protein solubilization were found by the end of small intestinal digestion among the pulses (90–95%).

**Readily bioaccessible protein:** The bioaccessible protein digestion kinetics revealed that by the beginning of the small intestinal phase less than 3% of the protein was bioaccessible (Figure 6A2,B2,C2). For all pulses, final protein digestion levels of 34–37% were reached. Estimated reaction rate constants of the readily bioaccessible protein fraction of CP and PE ICC were twice as high as for BB. Statistical analysis (Supplementary Materials Figure S1B) indicates that while estimated hydrolysis rates for the samples (CP and PE versus BB) differed, similar extents of protein digestion were obtained.

The difference between digested soluble and readily bioaccessible protein at the beginning of small intestinal digestion showed that about 23–34% of the protein was not yet readily bioaccessible. This indicates the presence of additional soluble fragments in the digestive supernatant, which would require further breakdown before being absorbed (i.e., brush border membrane). By the end of the small intestinal digestion, that fraction increased to 52–60%.

#### 3.3.4. Effects of Increased Microstructural Heterogeneity on Starch and Protein Digestion of Hardness-Aligned Pulses

Many scientists have applied a reductionist approach focusing on the ICC fraction when studying digestion barriers in pulses [14,15,18,21,23,24,26,32,33,50]. As anticipated, the microstructural characterization of both wet-sieved ICC and WSM exhibited differences in terms of structural heterogeneity witnessed by distinct PSD (unimodal versus multimodal) (Figure 5A–C) and microscopic observations Figure 5. To verify to what level the digestion kinetics of ICC characterizes the behavior of the WSM, starch and protein digestion kinetics of ICC and WSM were compared. Modeling of starch and protein digestion data showed similar digestion kinetics for the WSM and the respective ICC fraction (Section 3.3.2 and Section 3.3.3) (Table 2 and Table 3), except for starch digestion in CP WSM. For CP WSM, both, rate and extent of starch digestion were significantly lower.

### 3.4. Hydrothermal Processing Duration and Hardness as a Tool to Align In Vitro Macronutrient Digestion: The Case of Chickpeas and Black Beans

Hydrothermal processing times have been described to affect starch and protein digestion in pulses to different extents [4]. ICC isolated from BB and CP subjected to three distinct processing times achieving complete softening were used to study the effect of (hydrothermal) processing on starch and protein digestion. Based on observed differences in digestion kinetics of CP and PE versus BB, CP and BB were chosen as case studies. ICC were isolated after 30, 60, and 90 min for CP, and 60, 90, and 120 min for BB WSM, respectively.

#### 3.4.1. Characterization of ICC Fraction of Hardness-Aligned Pulses after Different Hydrothermal Treatment Times

Moisture, starch, and protein contents for ICC of BB and CP were similar for the three different processing times (Table 1). Figure 5D,E displays the increased yield of ICC as a function of hardness decrease with processing time for both WSM (CP: 39–41%; BB: 50–63%) (Figure 3). All processing times resulted in similar residual hardness levels (CP = 40 ± 1 N; BB = 42 ± 1 N) with distinct yields of ICC ranging from 39 up to 63% for both pulses. Whereas longer processing times of BB increased the ICC yields despite reaching the hardness plateau, this was not the case for CP.

#### 3.4.2. Starch Digestion of ICC from Different Hydrothermal Treatment Times

**Figure 8:**
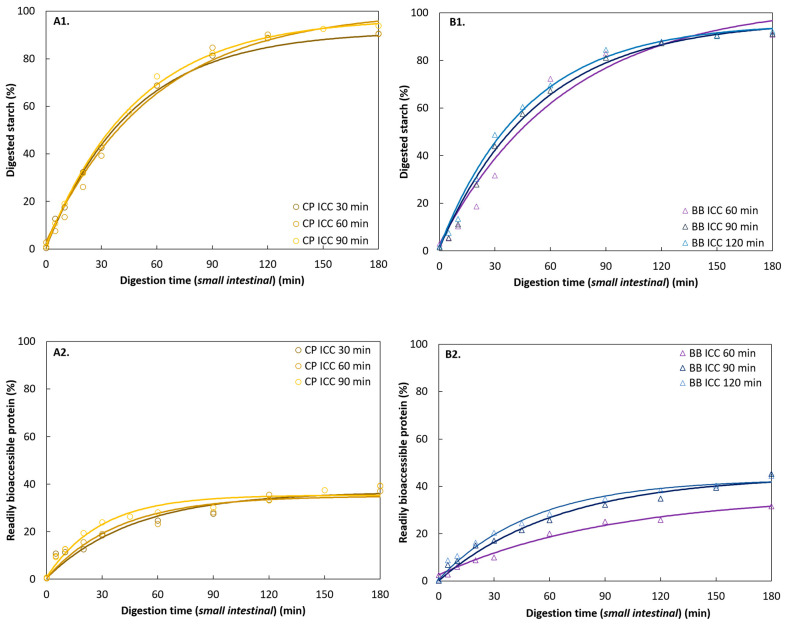
*In vitro* starch (**A1**,**B1**) and protein (**A2**,**B2**) small intestinal digestion kinetics of individual cotyledon cells (ICC) from chickpea (CP) (●) (**A**) or black beans (BB) (▲) (**B**) obtained after different hydrothermal treatment times (CP: 30, 60, or 90 min; BB: 60, 90, or 120 min). Symbols represent experimental values, while lines represent values predicted by a fractional conversion model (Equation (4)).

The starch digestion kinetics of ICC from BB and CP obtained after different processing times are presented in Figure 8. Experimental data showed a slower progression of starch digestion for all BB samples in the initial phase of digestion indicating the presence of a lag phase (t = 3–11 min) as described above (Section 3.3.2).

No significant differences between the estimated initial lag phases (λ) of the differently treated BB samples at the 95% confidence interval were found (Supplementary Materials Table S3). Nonetheless, it was clear that the duration of the lag phase followed a decreasing tendency with increasing processing times (BB60 = 11 min; BB90 = 5 min; BB120 = 3 min). In addition, modeling revealed that the reaction rate constant showed a reversed trend as presented by the lag phase and increased significantly with longer processing times. Regarding final starch digestion, significant differences between the shortest (60 min) and the longer processing times (90 and 120 min) were established. Increasing hydrothermal treatment times facilitated starch digestion in BB ICC (Table 4).

In contrast, starch digestion of the CP samples was characterized by an immediate highly dynamic phase (absence of lag phase). After 90 min, hydrolysis products stabilized and reached final starch digestion levels as previously described (Section 3.3.2). In contrast to BB samples, reaction rate constants of CP samples cooked for 30, 60, and 90 min did not statistically differ. However, the final starch digestion was significantly lower in the shortest cooking time (30 min).

#### 3.4.3. Protein Digestion of ICC from Different Hydrothermal Treatment Times

Similar to BB starch digestion kinetics, a trend of increasing estimated reaction rate constants and initial reaction rates with prolonged processing times was observed in BB (Table 5). The differences in the kinetic parameters suggest faster protein hydrolysis in ICC isolated after longer cooking times. Differences in final protein digestion levels (37–45%) were observed when comparing the shortest (BB60) versus longer processing times (BB90, BB120). Despite observable trends between all hydrothermal treatment times, especially hydrothermal treatment of 60 min or longer significantly increased protein digestion in BB. 

Equally, studying the protein digestion of CP samples revealed an increasing trend of reaction rate constants and initial reaction rates with hydrothermal processing times, although non-significant. Nonetheless, statistical analysis and modeling demonstrated that similar final protein digestion extents were obtained no matter the processing times.

### 3.5. Correlation between Starch and Protein Digestion in Pulses

A growing body of evidence exists that demonstrates the barrier effect of protein (digestion) to starch digestion [18,24,31,51].

#### 3.5.1. Effect of Pulse Type (Intrinsic Factors)

Correlations between the estimated, normalized levels of digested starch and protein were evaluated as a function of digestion time for ICC of CP, PE, and BB (Small box; Supplementary Materials Figure S2). A strong linear positive correlation between the level of digestion as a function of time in ICC of all three pulses could be observed. Nonetheless, minor differences in the correlations and thus different starch–protein digestion interdependencies were observed for the pulses. For the case of CP ICC, starch and protein digestion preceded linear, suggesting simultaneous starch and protein digestion. Contrary, for PE and BB, starch digestion seemed to be enhanced after 60% and 70% digestion, respectively. Looking at the normalized digestion data demonstrated protein preceding starch digestion in all samples. Statistical analysis and modeling indicate that protein digestion in the small intestinal phase generally proceeded faster than starch digestion (Table 3) except for BB.

Comparing the normalized data of WSM and ICC verified that the digestion behavior of ICC was representative for the WSM except for CP30. CP30 WSM showed an exponential correlation between estimated digested macronutrients. From these results, initially higher protein digestion was accompanied by faster protein and thus starch digestions. When looking at the WSM, the noticeable initial higher protein digestion levels, as compared to starch digestion, seem concomitant for higher starch digestion.

#### 3.5.2. Effect of Different Hydrothermal Processing Times (Extrinsic Factors)

The normalized data for the differently processed pulse samples revealed little differences in starch digestion with increasing processing time (data not shown). For protein, however, a clear trend of increased protein digestion with increasing processing time was observed for both pulse types as elaborated above (Section 3.4.3). Overall, modeling and statistical analysis suggest that starch digestion was little affected by hydrothermal processing times. Contrary, prolonged hydrothermal processing had a clear effect on the level of protein digestion, suggesting changes at the level of the intracellular protein rather than on the cell wall.

## 4. Discussion

In the present study, we intended to widen our investigation to the complementary analysis of the *in vitro* digestion behavior of both starch and protein in three pulses with distinct proximate and structural characteristics. We studied both the behavior of the WSM as well as ICC isolated thereof to establish process–structure–function relationships of hydrothermally treated pulses. To compare them on a more equivalent basis, hardness and hydrothermal processing were used as a tool to align microstructural characteristics.

### 4.1. Process-Induced Hardness Profile as a Material Property to Predict Microstructural Properties of Whole Pulses

In a first step, we wanted to study the suitability of process-induced hardness as a relatively easy-to-measure material property to obtain insights into the microstructural properties of pulse seeds. In this way, we wanted to consider a more equivalent basis (microstructure) to compare digestive properties among different pulse types. Therefore, hardness–microstructure relations as a function of cooking times were established (Figure 2 and Figure 3). Hardness profiles of BB indicated different softening susceptibility as compared to CP and PE, resulting in distinct time-dependent tissue failure modes. Fresh whole BB cooked slower than CP and PE, whereas CP and PE showed similar softening behavior (Figure 1). Nonetheless, final residual hardness levels were comparable for the pulses. These observations are in line with previously reported hardness changes [25] with longer softening times for kidney beans reported as compared to CP. As hardness degradation of pulses during cooking mostly involves simultaneous phenomena, namely starch gelatinization, and middle lamellae pectin solubilization with the latter being the rate-determining process [5], previous studies have linked different softening susceptibilities to intrinsic differences in dietary fiber, CW (polymer composition) and more specifically to differences in the amount and/or composition of middle lamellae pectin [25,52]. Specifically, fast cooking times for beans were associated with a lower dietary fiber content, thinner CW, and higher seed weight [52]. Under the premise that a thicker CW indicates a higher pectin content, hardness reduction of pulses with higher pectin content, as for BB, might be characterized by prolonged hydrothermal treatment times needed for complete softening. In this study, we have shown that the calculated FRR and CW thickness varied by pulse type, both being the highest in BB (Table 1, Figure 4) [53]. Accordingly, the higher FRR (Table 1) and CW thickness of BB might explain the prolonged cooking times and lower softening reaction rate constant (*k*) of BB as compared to CP and PE (Supplementary Materials Table S3). We hypothesize that faster pectin solubilization rates resulted in the obtainment of ICC at earlier cooking times in CP and PE as compared to BB (Figure 2).

Additionally, the different evaluations of particular CW properties (FFR, CW thickness) hint at a more pronounced CW barrier effect for digestion in BB compared to CP- and PE-related samples. Although pectin solubilization has been identified as the rate-limiting step for pulse tissue softening, starch gelatinization might partly play a role in fostering cell separation. In the case of sufficient pectin solubilization, it can be expected that a higher starch content, linked to a more pronounced starch swelling and gelatinization phenomena, might result in increased intracellular packing. This, together with the reduced strength of the CW as a result of pectin solubilization, potentially fostered cell separation in CP and PE. Therefore, in addition to the lower FRR fraction and CW thickness, the higher starch to FRR ratios (Table 1) observed for CP and PE might result in earlier cell separation and thus faster hardness reduction.

Other studies have shown that hardness profiles are affected by intrinsic factors, as well as extrinsic factors, among others by storage-induced changes [42] and cooking media [5]. Therefore, the authors would like to acknowledge that hardness profiles determined in this work are characteristic of the batches of pulses used in this work. 

Quantitative evaluation of the yield of ICC as a function of processing time highlighted the increase of ICC along with the decrease of hardness until reaching almost constant and maximal ICC yields (Figure 3). Within the residual hardness region of each pulse, similar microstructural properties could be observed, presumably independent of prolonged processing times. However, for BB, a slight increase of ICC yield with increasing processing times was observed, suggesting increased pectin solubilization in the middle lamella [5]. Our findings indicate that, apart from seed sizes and morphological characteristics [54], pulse-specific differences at the level of the CW influence predominantly hardness degradation kinetics, and by this, the opportunity to harvest ICC. This study revealed that aligning pulses at the level of their final residual hardness levels resulted in the prevalence of ICC and thus similar microstructural properties for BB, CP, and PE. This suggests that complete tissue softening, characterized by reaching residual hardness levels, could serve as an indicator for cell separation (tissue failure mode).

### 4.2. Structure-Aligned Pulses: The Consequences for Their In Vitro Macronutrient Digestion

#### 4.2.1. Comparative Study of Structure-Aligned Pulses: The Effect of Pulse Type

In pulses, microstructural characteristics play a key role during starch and protein gastrointestinal digestion. In this work, (residual) hardness was employed as a parameter to assess and subsequently align the microstructural properties of CP, PE, and BB using hydrothermal treatment. As elaborated before (Section 4.1), the alignment of residual hardness between the three pulses resulted in the alignment of their respective microstructural characteristics (ICC). The authors would like to note that process-induced changes at the level of the CW, which do not translate to changes in hardness (hardness plateau), cannot be excluded and were therefore further investigated (Section 4.2.2).

##### Starch Digestion

The retarded time-dependent behavior of *in vitro* starch digestion, which is characteristic for pulses and results from the macronutrient encapsulation, was observed for the three pulses as described previously by multiple authors [15,18,21,31]. The overall *in vitro* starch digestion kinetics of ICC of CP, PE, and BB were not influenced by pulse-specific factors after hardness-alignment. Hence, the structural alignment of the three pulse types resulted in similar starch digestion kinetics of ICC with minor differences during the initial phase of starch digestion (lag phase). With this, our results indicate that hydrothermal processing can be used to structurally align pulses and by this starch digestion kinetics. 

Apart from the overall observed similarities in the digestion kinetics, distinct but minor pulse-specific digestion behavior can be observed when zooming in into the initial digestion phase. Given the strong relationship of *in vitro* kinetic parameters (especially initial reaction rate constant) and the physiological relevance of those [55], understanding the nature of the pulse-specific influence on the initial digestion behavior is relevant. The assessment of the hydrolysis and initial reaction rate constant, being the lowest for BB, allowed for the identification of minor differences (presences versus absence of a lag phase) between CP and PE versus BB ICC. Lag phases, being linked to the digestive CW barrier effect, of varying times and processing sensitivities have been previously reported for starch digestion in other bean varieties (Canadian wonder 8 versus 35 min [21]) as well as for Bambara groundnuts (10 min) [24]. While this effect was observed for bean varieties, the absence of a lag phase was reported during starch digestion of ICC from CP, PE, and other pulses [32,56] and is consequently in line with our current observations. CW of different pulses have been reported to show variations in amount, composition [53,57], and natural and process-induced permeability/density [2,21,22,58]. Moreover, the relatively more densely protein-packed cytoplasmic matrix, indicated by a lower starch to protein ratio, potentially results in an initial delay of enzyme–substrate interactions until a sufficient level of concurrent protein digestion is reached. Along with this, other interactions between enzymes and food matrix components, such as the non-catalytic binding affinity to (soluble/insoluble) dietary fiber [26] and cellulose [59], have been found to reduce amylolytic activity. Another factor for the presence and/or absence of initial attenuated starch digestion kinetics could be the significantly smaller ICC size of BB, as compared to CP and PE, increasing the CW to enzyme ratio. Moreover, as demonstrated by the normalized digestion data (Supplementary Materials Figure S2), retarded starch digestion might be linked to the reduced level of gastric and/or simultaneous small intestinal protein digestion in BB ICC.

##### Protein Digestion

When looking at the implications of structure alignment on protein digestion, comparable protein digestion patterns were found for CP, PE, and BB ICC. As observed for starch digestion, differences in the initial minutes of the small intestinal digestion phase were observed. The reported ‘readily bioaccessible’ and ‘digested soluble’ protein extents are in line with previously reported ones for different legumes [18,24,25,31,46]. Apart from the lower reaction rate constant for BB60, no significant differences for both kinetic parameters between the structure-aligned pulses were found. In contrast to starch digestion, protein digestion was initiated during the gastric phase by the proteolytic action of pepsin. Gastric digestion led to 38% of total protein being digested for both BB and PE, while for CP 26% of the protein was solubilized upon entering small intestinal digestion. The differences in gastric proteolysis might cause faster protein digestion of CP as compared to PE and BB (Figure 6D; digested soluble). Overall, the absence of a pronounced lag phase in BB protein in contrast to starch digestion might be related to the smaller size and the lack of binding affinity of proteases to CW components as compared to amylases [58]. Our findings indicate overall slower protein digestion for BB, whereas no major differences between CP and PE proteolysis could be observed. Interestingly, (s)lower rates and levels of protein digestion of bean varieties (i.e., kidney bean [25], BB [60]) compared to other pulses, i.e., CP and PE, have been described in literature. Protein digestion in pulses has predominantly been linked to the effect of processing on protein structures [4,24] and little to be affected by processed-induced differences affecting enzymatic diffusion [24,31]. Processing has been described to render pulse protein either more susceptible or resistant for digestion, depending on the pulse type [4]. Intrinsic differences at the level of molecular amino acid sequence [61]. Differences in primary protein structure might result in different secondary or tertiary structures emerging in different affinities of proteolytic enzymatic attack and thus differences in digestion kinetics. Both processing history, as well as the botanical origin, might influence protein structure and, thus, denaturation and digestion kinetics [24,25,46,62]. Along with containing the highest levels of antinutrients (e.g., polyphenols) [63], BB are characterized by the lowest starch to protein ratio of the three investigated pulses. This might have implications on enzyme–substrate ratios, as aforementioned. Moreover, a link between protein digestion and antinutrient composition can be made. Several authors describe increased protein digestibility with decreasing antinutrient contents [64,65,66]. Thus, (s)lower protein digestion in BB might originate from the different intrinsic properties among the studied pulses.

Extending the comparison of hardness-aligned pulses to previous literature on pulses digestive behavior of similar hardness levels (40 N), similar microstructural properties, reaction rate constants and final starch and digested soluble protein levels could be observed in ICC of Bambara groundnuts treated for 120 min (Starch: *λ* = 10 min; *k_ma_*_x_ = 1.3 starch%/min; *C_f_* = 84%; Protein: DSP: *k*= 0.076 min^−1^; *C*_f_= 86%; BP: *k*= 0.043 min^−1^; *C*_f_= 57%) [24]. Looking at reported hardness–digestion relationships for common beans (∼45–60 min; 40 N), (s)lower starch digestion as compared to the ones reported in this study can be observed. Interestingly, looking at digestion kinetics of beans cooked to the residual hardness (~90 min; 25 N) [17], similar starch digestion behavior (*λ =* 16 min; *k_ma_*_x_ = 1.1 starch%/min; *C_f_* = 79%) [21] can be observed. One can hypothesize that prolonged softening times for common beans, as compared to the CP, PE, and BB, result in a delayed achievement of residual hardness due to similar reasons as elaborated for BB. That residual hardness is achieved after longer cooking times for BB signifies a broader window of hydrothermal treatment times. Thus, the period where more distinct process-induced, heterogeneous microstructures can be obtained until complete softening is achieved increases.

Our observations highlight that samples sharing similar structural properties show similar digestibility, despite differences in processing history and pulse type. With this, our results show that pulse digestion kinetics strongly depend on microstructural properties.

#### 4.2.2. Comparative Study of Structure-Aligned Pulses: The Effect of Processing Time and Resulting Microstructure

Current literature suggests that protein and starch digestion kinetics both depend on pulse type and processing method and/or intensity [4]. Next to the ambiguity of current findings, systematic and comparative studies investigating the effect of processing intensity simultaneously on starch and protein digestion of different pulses are missing. Increasing the understanding of the effect of the hydrothermal processing times on structure–digestion relation of different pulse types can be used to benefit from tailored starch and protein digestion behaviors to formulate processing strategies. Insights into process–structure–function relationships of pulses can be used not only for whole pulse processing, e.g., maintaining appropriated textures, but for ingredient manufacturing, e.g., increasing yield of ICC fraction. Therefore, our second aim was to evaluate the effect of prolonged hydrothermal processing times on starch and protein digestion in ICC isolated from CP and BB. Part of the selection criteria for the different processing times was to include equivalent hardness levels for the pulses for structural alignment (Section 3.4.1). In this case, ICC from pulses processed to final residual hardness levels for both CP and BB were chosen.

##### Starch Digestion

Interestingly, all samples exhibited similar final starch digestion extents (Table 4). For CP, the three samples demonstrated similar starch digestion behavior with comparable reaction rate constants and final digestion values (Figure 8A1,B1). These observations are in line with previous findings of our research unit, where starch digestion kinetics were similar for hardness-aligned Bambara groundnuts (~49 N), irrespective of processing time [24]. Contrary, an increase in starch digestion reaction rate constant with longer processing was observed for BB while reaching comparable final digestion extents. The influence of processing intensity on starch digestion has been investigated in detail for ICC from common beans [21]. Prolonged processing times were linked to increasing CW permeability due to process-induced changes at the level of pectin solubilization, and by this enabling the access to starch directly [58]. Besides, prolonged processing times can indirectly affect starch digestion by potentially increasing protein digestion during gastric and small intestinal digestion reducing the intracellular protein barrier [21]. As deduced from the PSD (Figure 3), the yield of BB ICC increased significantly with prolonged processing times. Moreover, the decreased lag phase with increasing processing times indicated that longer processing times resulted in greater access through the CW barrier. In addition, a decrease of the hardness variation upon prolonged processing could be observed by a smaller standard deviation, indicating decreased sample heterogeneity that is most likely caused by more homogeneous and increased pectin solubilization [5,24]. Thus, the increase of ICC yield for BB might hint at changes at the level of CW (polymers) with longer processing. Moreover, the increased protein digestion, and thus ‘reduced’ barrier, is likely a factor contributing to the increased starch accessibility.

##### Protein Digestion

The role of the protein barrier for starch digestion in pulses is well established [18]. Prolonged processing times affected protein digestion kinetics in the same way as they did for starch digestion (Figure 8A2,B2). While processing did not influence CP protein digestion, differences in BB protein digestion kinetics were observed (Table 5).

Several structural factors can play a role in increasing protein digestion upon longer hydrothermal treatment in BB as recently reviewed by Duijsens et al. [4]. In this study, most likely a combination of structural changes at the level of CW and intracellular protein matrix were responsible for the process-induced increased protein digestion. On the one hand, changes at the level of CW permeability, witnessed by increased ICC yield (microstructural changes) and the decreased lag phase, could have a positive effect on gastric protein digestion resulting in differences at the level of protein barrier when starch digestion is initiated (small intestinal digestion). On the other hand, increased processing times are likely to increase protein denaturation, promoting protein unfolding, and thus the exposure of hidden α-amino groups [18,67,68,69]. To sum up, in the case of ICC from BB within the residual hardness range, process-induced CW permeability did affect *in vitro* starch and protein digestion. We hypothesize that processing increased pectin solubilization even in the hardness plateau resulting in CW changes. However, this was not the case for CP samples, where CW and/or cytoplasmic matrix constitute a barrier after thermal treatment, which was little further affected by prolonged processing.

Nonetheless, the authors would like to note that despite the elaborated reasoning the process-induced effects for ICC from BB seem minor, especially when compared to previously reported effects [21,24]. Our results suggest that at the level of the ICC from different pulses, hardness can be used to standardize structural properties and by this starch and protein digestion. However, this systematic slower protein digestibility observed in beans might be interesting to investigate further. This might be especially interesting in the context of looking at the co-ingestion of starch and protein and/or trying to enhance nutritional properties by removal of antinutritional factors (e.g., enzymatic pre-treatment).

### 4.3. Effect of Increased Microstructural Heterogeneity on the Digestive Response (ICC versus WSM)

Insights on the digestive response of pulses are mainly generated using a reductionist approach investigating the ICC of pulses exclusively after dehulling and wet-sieving [3]. Besides, inherently innovative processing of pulses to ICC-rich ingredients results in by-products consisting of high levels of dietary fiber, minerals, and potential phytochemicals [70]. Against the background of exploring the potential use of whole hydrothermally treated pulse flours, our third objective was to investigate the digestive behavior of the WSM along with its respective isolated ICC fraction (Figure 6). Overall, starch and protein digestion kinetics of pulses’ ICC were representative for the digestive behavior of the WSM with one exception: the WSM of CP showed significantly (s)lower starch digestion than its ICC. Once more, this could be explained considering the microstructural characteristics of the WSM and the respective ICC fraction. Wet-sieving of the WSM resulted in a decrease of microstructural heterogeneity for the ICC (from multimodal to unimodal) (Figure 5), wherein the WSM comprised 38%, 38%, and 50% ICC, for CP, PE, and BB, respectively. The width of the PSD of CP WSM, showing the lowest and broadest abundance of particles sizes in the range around 100 µm, might hint at higher microstructural heterogeneity as compared to BB and PE. The differences between the PSD of CP WSM and the ICC fraction imply a larger structural discrepancy between the two sample types, WSM and ICC, as compared to PE and BB. Additionally, a higher level of microstructural heterogeneity in the WSM increases the amount of soluble material which might result in increased crowding and bulking. Thereby, decreased enzyme–substrate interaction can be anticipated due to depletion and spacial hindrance [71,72]. Moreover, CW material and dietary fiber have been described to delay starch digestion through non-specific binding of, i.e., cellulose and other insoluble fibers to α-amylase [14,15,26]. Water-soluble fibers are capable of inhibiting α-amylase activity directly following a different mechanism [73]. While seed coat to cotyledon ratios (Table 1) were not significantly different for the pulses, the compositional difference at the level of soluble dietary fiber, being higher for CP than for PE and BB [53], might impact the digestive behavior of CP WSM. Therefore, we can only hypothesize that the (s)lower starch hydrolysis of the CP WSM might originate from a combination of non-specific binding/adsorption interactions and microstructural differences between ICC and WSM of hydrothermally treated CP. These have been described for starch degrading enzymes, however, not when looking at proteases. This might explain the absence of significantly different rates or extents of protein digestion. These observations highlight that in the case of ICC of PE and BB processed to a residual hardness level, the most abundant microstructure (ICC) is likely to control *in vitro* starch and protein digestion kinetics. We believe these observations suggest that digestive insights generated on ICC or WSM might be transferable. This offers the possibility for increased knowledge transfer of the current research. More strikingly, these insights offer great industrial/application possibilities for the generation of innovative, whole pulse-based flours. The incorporation of the seed coat fraction into the pulses and thus food products might result in decreased waste streams.

On a different note, the qualitative and quantitative analyses of *in vitro* starch and protein digestion have revealed the presence of undigested material in pulse cotyledon cells after simulated small intestinal conditions. Thus, the investigation of the remaining pellets during colonic fermentation in the large intestine would be a significant next step to take, along with the assessment of soluble and insoluble dietary fiber fractions. Not only the potential of these microstructures as delivering systems for the microbiota, but the role of the residual cellular microstructure on the actual starch degradation as well as on the degradation of other nutrients by the microbiota would be insightful.

## 5. Conclusions

Hydrothermal processing to equivalent hardness levels was proven to be an effective tool to generate (micro)structures with similar susceptibility to *in vitro* starch and protein hydrolysis for three pulse types. Our findings have illustrated that although pulses responded with distinct hardness degradation kinetics to hydrothermal processing, similar residual hardness levels were reached upon prolonged processing times. Individual cotyledon cells are the characteristic microstructure after mechanical disintegration of whole pulses processed to residual hardness. Reaching these plateau-hardness levels indicates pronounced levels of pectin solubilization resulting in cell separation and can thus be linked to high abundances of ICC. In addition, the evaluation of ICC from chickpeas, pea, and black beans processed to equivalent residual hardness levels demonstrated that digestion kinetics strongly depend on microstructural properties governed by processing sensitivity. Furthermore, prolonged processing times applied to hardness-aligned chickpea and black beans samples did not significantly affect digestion kinetics. Our results highlight the suitability of hardness as a material property to align structural properties and by this digestion kinetics between pulses. Comparing the digestive behavior of the characteristic microstructure (ICC) to its respective whole pulse material did not reveal pronounced differences. These insights offer the possibility of using whole pulses rather than ICC for future innovative food design. The use of the whole, hydrothermally pre-treated pulse flours would offer the incorporation of dietary fiber, minerals, and potential phytochemicals while at the same time avoiding the production of by-products.

Nonetheless, minor pulse-specific behavior was still observed when looking at the rate of hardness decay as well as the initial phase of starch digestion behavior, especially pronounced in beans. The different sensitivity to hydrothermal treatment may originate from differences in CW composition and process-induced permeability. Additional research focusing on the characterization of CW properties of pulses to unravel what makes beans different from other pulses is needed.

To conclude, this study expanded the knowledge on the effect of hydrothermal processing on pulses’ digestive responses. These insights can be used in the context of targeted nutrition and the optimization of processing procedures. We suggest taking the next step in the use of pulse-based ingredients/flours by using whole fractions of thermally treated pulse seeds characterized by high levels of digestive barriers, rather than using ICC fractions isolated thereof or conventionally processed flours with broken cells.

## Figures and Tables

**Table 1:** Proximate composition ± standard deviation of raw (per 100 g ingredient) and hydrothermally processed (per 100 g dry matter (dm)) chickpea (CP), pea (PE), or black bean (BB) seeds. Mean values (within a column) with different superscript letters are significantly different (α = 0.05). The composition of isolated cotyledon cell fractions (ICC), as well as for the whole seed material (WSM) of chickpea, pea, or black bean, are reported for the hydrothermally treated pulses (95 °C, t (min)).

Sample	Total Starch (g/100 g)	Total Protein (N*5.4) (g/100 g)	Total Non- starch Lipids (g/100 g)	Ash (g/100 g)	Fiber-Rich Residue (g/100 g Calculated)	Moisture (g/100 g)	Starch–Protein Ratio	Starch–Fiber-Rich Residue Ratio	Seed Coat–Cotyledon Ratio (*w*/*w*)
**CP raw**	39.73 ± 1.61 ^a^	19.39 ± 0.12 ^a^	7.29 ± 0.28 ^a^	2.17 ± 0.40 ^a^	27.53 ^a^	3.89 ± 0.17 ^a^	2.05	1.44	0.14
**PE raw**	40.90 ± 1.42 ^a^	19.73 ± 1.10 ^a^	2.79 ± 0.10 ^b^	1.87 ± 0.28 ^a^	27.29 ^a^	7.43 ± 0.28 ^b^	2.07	1.50	0.15
**BB raw**	33.34 ± 1.72 ^c^	20.64 ± 0.53 ^a^	2.45 ± 0.39 ^b^	3.70 ± 0.28 ^b^	32.63 ^a^	7.23 ± 0.03 ^b^	1.62	1.02	0.11
**Sample**		**Total Starch** **(g/100 g dm)**		**Total Protein** (N*5.4) (g/100 g dm)		**Starch–Protein Ratio**		**Moisture (g/100 g)**	
**CP WSM 30 min**		41.24 ± 5.92 ^b^		19.65 ± 0.21 ^a^		2.10		64.42 ± 0.75 ^a^	
**PE WSM 30 min**		45.48 ± 3.19 ^a^		17.60 ± 0.56 ^b^		2.58		62.57 ± 4.52 ^a^	
**BB WSM 60 min**		38.61 ± 1.20 ^b^		18.67 ± 0.27 ^a,b^		2.07		62.66 ± 0.19 ^a^	
**Sample**		**Total Starch** **(g/100 g dm)**		**Total Protein** (N*5.4) (g/100 g dm)		**Starch–Protein Ratio**		**Moisture (g/100 g)**	
**PE ICC 30 min**		61.21 ± 1.22 ^a^		17.74 ± 0.06 ^c^		3.45		74.45 ± 0.11 ^a^	
**CP ICC 30 min**		54.96 ± 0.64 ^b^		16.94 ± 0.02 ^e^		3.24		74.24 ± 0.25 ^a^	
**CP ICC 60 min**		55.33 ± 2.65 ^b^		16.57 ± 0.02 ^e^		3.34		81.17 ± 1.14 ^a,b^	
**CP ICC 90 min**		54.56 ± 2.65 ^b^		17.33 ± 0.11 ^c,e^		3.15		81.31 ± 0.55 ^a,b^	
**BB ICC 60 min**		47.64 ± 2.95 ^c^		19.98 ± 0.13 ^b^		2.38		73.19 ± 0.02 ^b^	
**BB ICC 90 min**		51.31 ± 1.68 ^b,c^		21.06 ± 0.23 ^b^		2.44		82.28 ± 1.75 ^a^	
**BB ICC 120 min**		50.44 ± 0.01 ^b,c^		22.10 ± 0.01 ^a^		2.28		82.10 ± 0.01 ^a^	

**Table 2:** Estimated kinetic parameters ± standard deviation of *in vitro* starch digestion of individual cotyledon cells (ICC) and whole pulse seeds (WSM) from chickpea (CP), pea (PE), or black bean (BB) aligned to equivalent hardness levels using hydrothermal treatment for 30, 30, or 60 min, respectively. Starch digestion kinetic parameters were estimated by fitting the experimental data to a fractional conversion model (Equation (4)). Mean values (within a column) with different superscript letters are significantly different based on 95% confidence intervals.

Sample	*In Vitro* Starch Digestion Kinetics (Small Intestinal)			
	*k* (min^−1^)	*Starch_f_* (%)	Initial Reaction Rate (% × min^−1^)	R^2^ _adjusted_
**CP ICC 30 min**	0.018 ± 0.001 ^a^	91.67 ± 1.63 ^a^	1.96 ± 0.10 ^a^	0.99
**PE ICC 30 min**	0.015 ± 0.002 ^a^	88.75 ± 4.68 ^a,b^	1.52 ± 0.22 ^b^	0.99
**BB ICC 60 min**	0.014 ± 0.004 ^a^	104.00 ± 11.02 ^a,b^	1.48 ± 0.39 ^b,d^	0.99
**CP WSM 30 min**	0.023 ± 0.002 ^a^	84.37 ± 3.89 ^a,b^	1.06 ± 0.13 ^c^	0.99
**PE WSM 30 min**	0.019 ± 0.001 ^a^	82.09 ± 1.46 ^b^	1.72 ± 0.09 ^b,d^	0.99
**BB WSM 60 min**	0.018 ± 0.003 ^a^	93.61 ± 5.06 ^a,b^	1.66 ± 0.25 ^b^	0.99

**Table 3:** Estimated kinetic parameters ± standard deviation of *in vitro* protein digestion of individual cotyledon cells (ICC) and whole pulse seeds (WSM) from chickpea (CP), pea (PE), or black bean (BB) aligned to equivalent hardness levels using hydrothermal treatment for 30, 30, or 60 min, respectively. Protein digestion kinetic parameters were estimated by fitting the experimental data to a fractional conversion model (Equation (4)). Mean values (within a column) with different superscript letters are significantly different based on 95% confidence intervals.

Sample	*In Vitro* Protein Digestion Kinetics (Small Intestinal)							
	Readily Bioaccessible Protein				Digested Soluble Protein			
	*k* (min^−1^)	*Protein_f_* (%)	Initial Reaction Rate (% × min^−1^)	R^2^_adjusted_	*k* (min^−1^)	*Protein_f_* (%)	Initial Reaction Rate (% × min^−1^)	R^2^_adjusted_
**CP ICC 30 min**	0.021 ± 0.005 ^a,b^	36.84 ± 3.46 ^a,b^	0.77 ± 0.21 ^a^	0.98	0.039 ± 0.004 ^a^	88.73 ± 1.83 ^a^	2.51 ± 0.24 ^a^	0.99
**PE ICC 30 min**	0.027 ± 0.004 ^a^	34.35 ± 1.44 ^a^	0.84 ± 0.12 ^a^	0.99	0.031 ± 0.004 ^a^	95.50 ± 2.20 ^a^	1.84 ± 0.22 ^b^	0.99
**BB ICC 60 min**	0.015 ± 0.002 ^b^	36.68 ± 3.11 ^a,b^	0.36 ± 0.07 ^b^	0.99	0.016 ± 0.002 ^b^	94.22 ± 4.03 ^a^	0.94 ± 0.15 ^c^	0.99
**CP WSM 30 min**	0.027 ± 0.006 ^a,b^	35.83 ± 2.30 ^a^	0.82 ± 0.19 ^a^	0.99	n.d	n.d	n.d	n.d
**PE WSM 30 min**	0.026 ± 0.006 ^a,b^	37.78 ± 2.42 ^a^	0.78 ± 0.19 ^a^	0.99	n.d	n.d	n.d	n.d
**BB WSM 60 min**	0.022 ± 0.001 ^a,b^	26.10 ± 1.69 ^b^	0.46 ± 0.04 ^b^	0.99	n.d	n.d	n.d	n.d

n.d: Not detected.

**Table 4:** Estimated kinetic parameters ± standard deviation of *in vitro* starch digestion of individual cotyledon cells (ICC) from chickpea (CP) or black bean (BB) aligned to equivalent hardness levels using hydrothermal treatment for 30, 60, or 90 min and 60, 90, or 120 min, respectively. Starch digestion kinetic parameters were estimated by fitting the experimental data to a fractional conversion model (Equation (4)). Mean values (within a column) with different superscript letters are significantly different based on 95% confidence intervals.

Sample	*In Vitro* Starch Digestion Kinetics (Small Intestinal)			
	*k* (min^−1^)	*Starch_f_* (%)	Initial Reaction Rate (% × min^−1^)	R^2^_adjusted_
**CP ICC 30 min**	0.021 ± 0.001 ^a^	91.67 ± 1.63 ^a^	1.96 ± 0.10 ^a^	0.99
**CP ICC 60 min**	0.017 ± 0.002 ^a^	100.30 ± 5.35 ^b^	1.67 ± 0.24 ^b^	0.99
**CP ICC 90 min**	0.020 ± 0.001 ^a^	97.17 ± 1.20 ^b^	1.97 ± 0.07 ^a^	0.99
**BB ICC 60 min**	0.015 ± 0.003 ^a^	104.00 ± 11.02 ^a,b^	1.48 ± 0.39 ^b^	0.99
**BB ICC 90 min**	0.019 ± 0.001 ^a^	96.68 ± 2.94 ^b^	1.78 ± 0.15 ^b^	0.99
**BB ICC 120 min**	0.021 ± 0.001 ^a^	95.58 ± 2.54 ^b^	1.97 ± 0.15 ^a^	0.99

**Table 5:** Estimated kinetic parameters ± standard deviation of *in vitro* protein digestion of individual cotyledon cells (ICC) from chickpea (CP) or black bean (BB) (aligned to equivalent hardness levels using hydrothermal treatment for 30, 60, or 90 min and 60, 90, or 120 min, respectively. Protein digestion kinetic parameters were estimated by fitting the experimental data to a fractional conversion model (Equation (4)). Mean values (within a column) with different superscript letters are significantly different based on 95% confidence intervals.

Sample	*In Vitro* Readily Bioaccessible Protein Digestion Kinetics (Small Intestinal)			
	*k* (min^−1^)	*Protein_f_* (%)	Initial Reaction Rate (% × min^−1^)	R^2^_adj_
**CP ICC 30 min**	0.021 ± 0.005 ^a,b^	36.84 ± 3.46 ^a^	0.77 ± 0.21 ^a^	0.98
**CP ICC 60 min**	0.026 ± 0.006 ^a^	35.05 ± 2.82 ^a^	0.90 ± 0.22 ^a^	0.98
**CP ICC 90 min**	0.034 ± 0.005 ^a^	35.43 ± 1.50 ^a^	1.18 ± 0.18 ^b^	0.99
**BB ICC 60 min**	0.011 ± 0.002 ^b^	36.68 ± 3.11 ^a^	0.36 ± 0.07 ^c^	0.99
**BB ICC 90 min**	0.015 ± 0.002 ^a,b^	44.70 ± 2.71 ^a^	0.68 ± 0.10 ^a^	0.99
**BB ICC 120 min**	0.020 ± 0.002 ^a,b^	43.13 ± 1.81 ^a^	0.84 ± 0.10 ^a^	0.99

## Data Availability

The data presented in this study are openly available on Zenodo.org at 10.5281/zenodo.5834249.

## Supplementary Materials

The following are available online at https://www.mdpi.com/article/10.3390/foods11020206/s1, Figure S1: Joint confidence region (JCR) (95% confidence level) of the estimated fractional conversion model kinetic parameters for the *in vitro* starch (A) and protein (B) hydrolysis of hardness-aligned individual cotyledon cells (ICC) of chickpea (●), pea (■) or black bean (▲) samples hydrothermally processed for 30, 30 or 60 min respectively. Markers in JCR represent the estimated rate constant and final digested starch or protein for each digestion. Figure S2: Normalized parameter estimates of *in vitro* starch (line) and readily bioaccessible protein (dotted line) small intestinal digestion kinetics of individual cotyledon cells (ICC) (A1–C1) and whole seed material (WSM) (A2–C2) from hardness-aligned chickpea (●) (A), pea (■) (B) and black bean (▲) (C) hydrothermally processed for 30, 30 or 60 min respectively. Starch and protein digestion kinetic parameters were estimated by fitting the experimental data to a fractional conversion model (Equation (4)). Scatter plots within the normalized digestion plots show correlation analysis of starch versus protein digestion parameters of hydrothermally processed pulses,. Table S1: Parameter estimates ± standard errors for the softening of whole chickpea, pea or black bean seeds cooked in demineralized water at 95 °C. For this, the fractional conversion model (Equation (4)) was fitted to the experimentally determined hardness values. Absolute hardness values represent mean values of hardness measurements from 25 cotyledons in Newton (N). Relative hardness values are expressed as the ratio of initial hardness (H_i_) in percent (%). *k*-values and *H_f_* having different superscript letters are significantly different based on 95% confidence intervals., Table S2: Differential scanning calorimetry peak temperatures (Tp) and enthalpies of individual cotyledon cells (ICC) from hardness-aligned chickpea (CP), pea (PE) and black bean (BB) hydrothermally processed for 30, 30 or 60 min respectively. Each row gives means and standard deviations of means of duplicate measurements., Table S3: Estimated kinetic parameters ± standard deviation of *in vitro* starch digestion of individual cotyledon cells (ICC) from black bean (BB) aligned to equivalent hardness level obtained after different hydrothermal processing times. Starch digestion kinetic parameters were estimated by fitting the experimental data to a logistic model (Equation (6)). Mean values (within a column) with different superscript letters having different superscript letters are significantly different based on 95% confidence intervals.

## Abbreviations

BB—black bean; CP—chickpea; CW—cell wall; DM—dry matter; FRR—fiber-rich residue; ICC—individual cotyledon cells; JCR—joint confidence region; PE—pea; PSD—particle size distribution; TCA—trichloroacetic acid; WSM—whole seed material.
