# Supplementary material for: Utilizing Hydrothermal Processing to Align Structure and In Vitro Digestion Kinetics between Three Different Pulse Types
Source: Foods. 2022 Jan 12;11(2):206. doi: 10.3390/foods11020206 (PMC8775171; doi:10.3390/foods11020206)
Supplement: Supplementary file 1 [file foods-11-00206-s001.zip › foods-1442560-supplementary-final-118.pdf]

Supplementary Materials

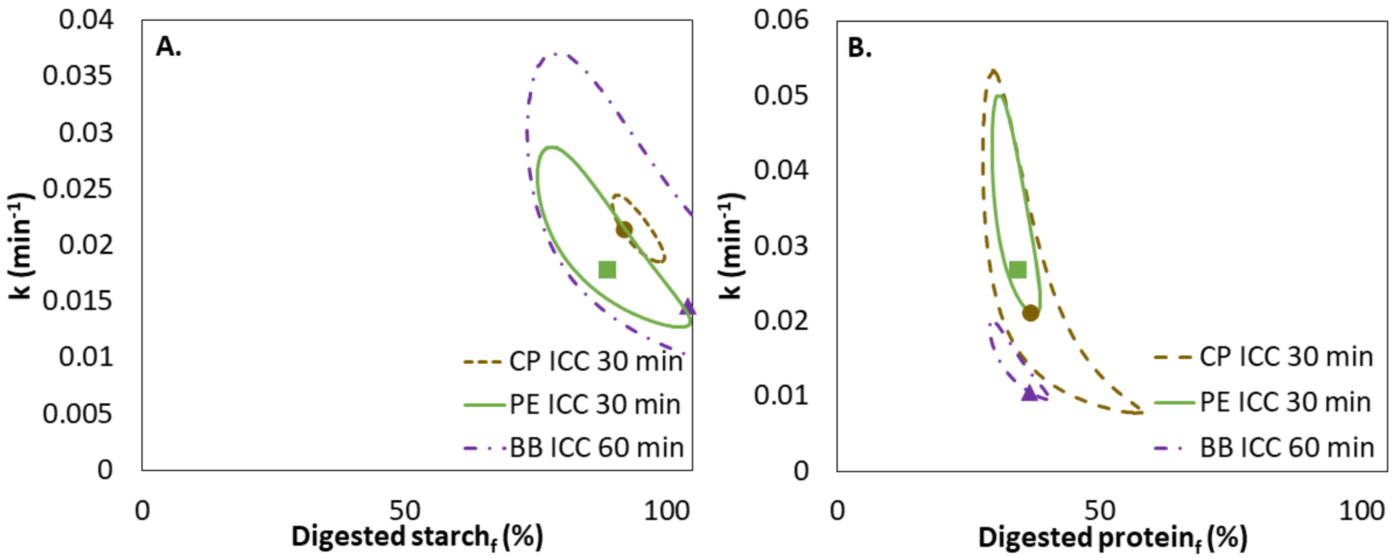

Supplementary Material Figure S1. Joint confidence region (JCR) (95% confidence level) of the estimated fractional conversion model kinetic parameters for the *in vitro* starch (A) and protein (B) hydrolysis of hardness-aligned individual cotyledon cells (ICC) of chickpea (CP) (●), pea (PE) (■) or black bean (BB) (▲) samples hydrothermally processed for 30, 30 or 60 min respectively. Markers in JCR represent the estimated rate constant and final digested starch or protein for each digestion.

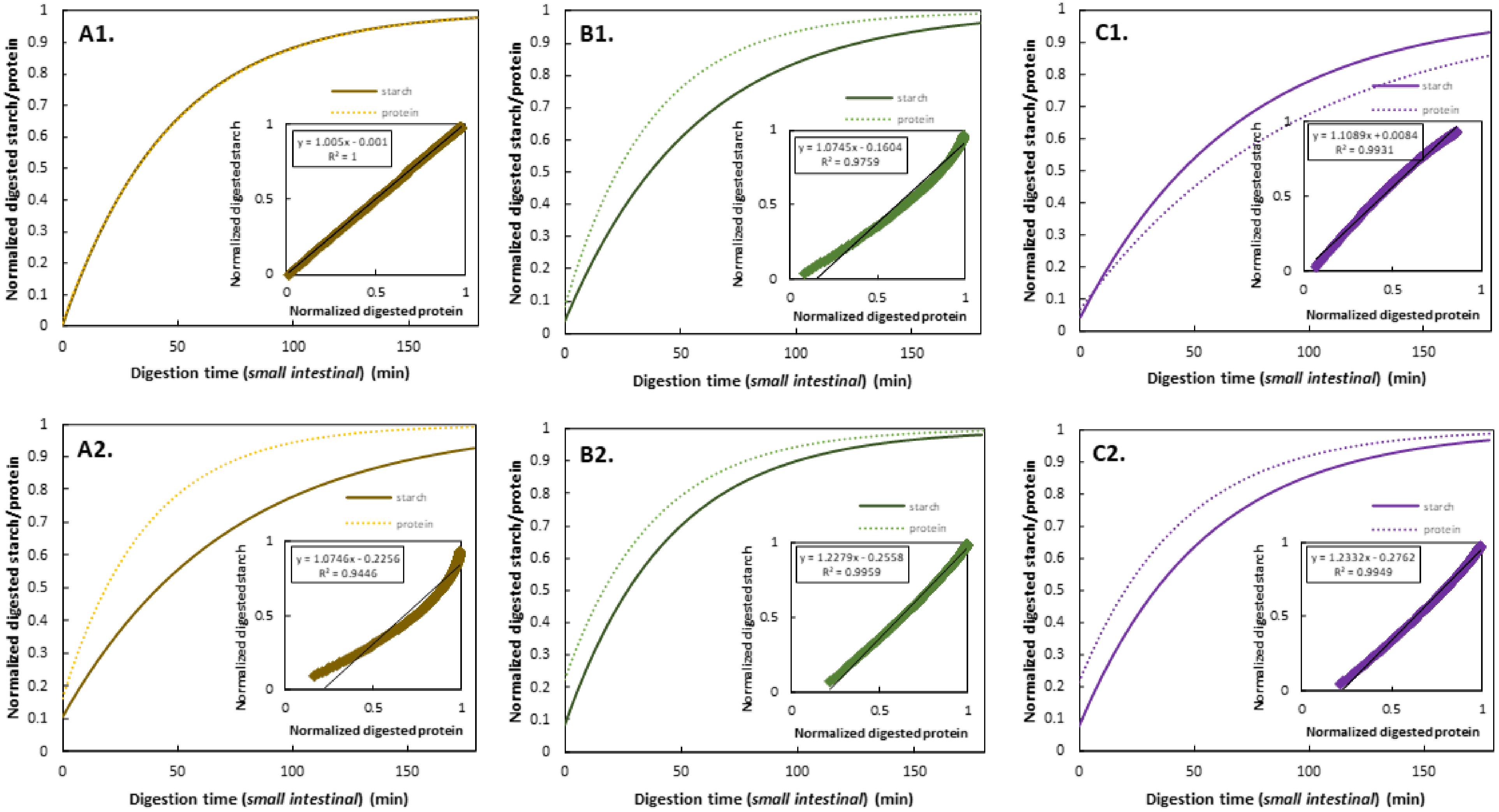

Supplementary Material Figure S2. Normalized parameter estimates of *in vitro* starch (line) and readily bioaccessible protein (dotted line) small intestinal digestion kinetics of individual cotyledon cells (ICC) (A1-C1) and whole seed material (WSM) (A2-C2) from hardness-aligned chickpea (●) (A), pea (■) (B) and black bean (▲) (C) hydrothermally processed for 30, 30 or 60 min respectively. Starch and protein digestion kinetic parameters were estimated by fitting the experimental data to a fractional conversion model (Equation 4). Scatter plots within the normalized digestion plots show correlation analysis of starch versus protein digestion parameters of hydrothermally processed pulses.

Supplementary Material Table S1. Parameter estimates ± standard errors for the softening of whole chickpea, pea or black bean seeds cooked in demineralized water at 95 °C. For this, the fractional conversion model (Equation 4) was fitted to the experimentally determined hardness values. Absolute hardness values represent mean values of hardness measurements from 25 cotyledons in Newton (N). Relative hardness values are expressed as the ratio of initial hardness (H_i) in percent (%). k-values and H_f having different superscript letters are significantly different based on 95% confidence intervals.

Sample	Absolute hardness			Relative hardness		
	k (min^{-1})	H_f (N)	$R^2_{adjusted}$	k (min^{-1})	H_f (%)	$R^2_{adjusted}$
**Chickpea**	0.199 ± 0.010 [a]	39.78 ± 0.94 [a]	0.982	0.199 ± 0.010 [a]	0.29 ± 0.01 [a]	0.982
**Pea**	0.161 ± 0.008 [b]	45.14 ± 0.96 [b]	0.984	0.161 ± 0.008 [b]	0.33 ± 0.01 [b]	0.984
**Black bean**	0.046 ± 0.002 [c]	42.25 ± 1.32 [a, b]	0.989	0.046 ± 0.002 [c]	0.28 ± 0.01 [a]	0.989

Supplementary Material Table S2. Differential scanning calorimetry peak temperatures (Tp) and enthalpies of individual cotyledon cells (ICC) from hardness-aligned chickpea (CP), pea (PE) and black bean (BB) hydrothermally processed for 30, 30 or 60 min respectively. Each row gives means and standard deviations of means of duplicate measurements.

Sample	Tp (°C)	Enthalphy (J/g)
**CP ICC 30 min**	57.89 ± 0.71 [a]	0.079 ± 0.009 [a]
**PE ICC 30 min**	58.92 ± 0.09 [a]	0.125 ± 0.018 [b]
**BB ICC 60 min**	59.83 ± 0.26 [a]	0.251 ± 0.056 [c]

Supplementary Material Table S3. Estimated kinetic parameters ± standard deviation of in vitro starch digestion of individual cotyledon cells (ICC) from black bean (BB) aligned to equivalent hardness level obtained after different hydrothermal processing times. Starch digestion kinetic parameters were estimated by fitting the experimental data to a logistic model (Equation 6). Mean values (within a column) with different superscript letters having different superscript letters are significantly different based on 95% confidence intervals.

Sample	***In Vitro* Starch Digestion Kinetics (Small Intestinal)**			
	Lag λ **(min)**	k_{max} **(starch%/min)**	**Starch$_{final}$ (%)**	R^2_{adj}
**BB ICC 60 min**	11.03 ± 1.41[a]	1.59 ± 0.09 [a]	87.91 ± 1.29 [a]	0.99
**BB ICC 90 min**	4.63 ± 3.85 [a]	1.44 ± 0.18 [a]	87.41 ± 2.82 [a]	0.99
**BB ICC 120 min**	3.01 ± 3.70 [a]	1.51 ± 0.19 [a]	88.00 ± 2.77 [a]	0.99
